# Genome-Wide Analysis of Adaptive Molecular Evolution in the Carnivorous Plant *Utricularia gibba*

**DOI:** 10.1093/gbe/evu288

**Published:** 2015-01-09

**Authors:** Lorenzo Carretero-Paulet, Tien-Hao Chang, Pablo Librado, Enrique Ibarra-Laclette, Luis Herrera-Estrella, Julio Rozas, Victor A. Albert

**Affiliations:** ^1^Department of Biological Sciences, University at Buffalo; ^2^Departament de Genètica and Institut de Recerca de la Biodiversitat (IRBio), Universitat de Barcelona, Spain; ^3^Laboratorio Nacional de Genómica para la Biodiversidad-Langebio/Unidad de Genómica Avanzada UGA, Centro de Investigación y Estudios Avanzados del IPN, Irapuato, Guanajuato, México; ^4^Present address: Red de Estudios Moleculares Avanzados, Instituto de Ecología A.C., Xalapa, Veracruz, México

**Keywords:** genome evolution, phenotypic diversification, carnivorous plants, gene family expansions and contractions, positive selection

## Abstract

The genome of the bladderwort *Utricularia gibba* provides an unparalleled opportunity to uncover the adaptive landscape of an aquatic carnivorous plant with unique phenotypic features such as absence of roots, development of water-filled suction bladders, and a highly ramified branching pattern. Despite its tiny size, the *U. gibba* genome accommodates approximately as many genes as other plant genomes. To examine the relationship between the compactness of its genome and gene turnover, we compared the *U. gibba* genome with that of four other eudicot species, defining a total of 17,324 gene families (orthogroups). These families were further classified as either 1) lineage-specific expanded/contracted or 2) stable in size. The *U. gibba*-expanded families are generically related to three main phenotypic features: 1) trap physiology, 2) key plant morphogenetic/developmental pathways, and 3) response to environmental stimuli, including adaptations to life in aquatic environments. Further scans for signatures of protein functional specialization permitted identification of seven candidate genes with amino acid changes putatively fixed by positive Darwinian selection in the *U. gibba* lineage. The *Arabidopsis* orthologs of these genes (*AXR*, *UMAMIT41*, *IGS*, *TAR2*, *SOL1, DEG9*, and *DEG10*) are involved in diverse plant biological functions potentially relevant for *U. gibba* phenotypic diversification, including 1) auxin metabolism and signal transduction, 2) flowering induction and floral meristem transition, 3) root development, and 4) peptidases. Taken together, our results suggest numerous candidate genes and gene families as interesting targets for further experimental confirmation of their functional and adaptive roles in the *U. gibba*’s unique lifestyle and highly specialized body plan.

## Introduction

The carnivorous syndrome has evolved multiple times among the flowering plants as a specialized solution for nutrient acquisition in phosphorus- and nitrogen-limited environments (Ellison and Gotelli 2009). Although the fascinating trapping strategies and specialized body designs of carnivorous plants have attracted scientific interest for centuries (Darwin 1875), the underlying genetic basis of the carnivorous syndrome remains largely unknown. The asterid family Lentibulariaceae is the largest and most phenotypically diverse carnivorous plant family, comprising three genera with distinct trapping mechanisms: *Pinguicula*, *Genlisea,* and *Utricularia* (Juniper et al. 1989; Taylor 1989). *Utricularia*, the bladderworts, are the largest and most diverse of all carnivorous plants. *Utricularia* species typically live in nutrient-poor aquatic, terrestrial, or epiphytic environments (Chao 2003), where they supplement normal photolithotrophic nutrition by trapping various microscopic prey animals (Givnish 1989). The trapping structures of *Utricularia*, the bladder-like suction traps, rank among the most complex leaf structures known in the plant kingdom (Juniper et al. 1989). These bladders can appear on apparently dissimilar parts of the plant body in different species, and their morphologies are diverse. Other than concerning the traps, a single *Utricularia* body plan is difficult to define; the boundaries between classical organ identities are blurred to the extent that leaf versus shoot becomes a difficult diagnosis for many species, especially given their lack of true roots (Adlassnig et al. 2005).

The recent publication of the nuclear genome of *Utricularia gibba*, an obligate aquatic bladderwort with thread-like and highly ramified vegetative organs, has revealed several interesting genomic architectural features (Ibarra-Laclette et al. 2013). Interestingly, the tiny *U. gibba* genome (82 Mb) results from the shrinkage of noncoding DNA, and not from an appreciable reduction in overall gene number (Ibarra-Laclette et al. 2013). Despite this comparable gene number, *U. gibba* has undergone three whole-genome duplication (WGD) events since its common ancestry with grape (Ibarra-Laclette et al. 2013). As a result, vast genetic resources were introduced to its genome, leading to multiple opportunities for possible functional specialization of gene duplicates. Therefore, it is expected that gene turnover has had an impact on *U. gibba*’s unique lifestyle, especially when its present tiny genome size suggests a strong constraint on its genetic content.

The evolution and architecture of plant genomes is greatly shaped by recurrent events of gene duplication and loss events (Lynch and Conery 2000; Blanc and Wolfe 2004; Cui et al. 2006; Fei et al. 2006; Jiao et al. 2011; Proost et al. 2011), which are believed to play a key role in generating plant phenotypic diversity and speciation, including the explosive early diversification of angiosperms (De Bodt et al. 2005; Soltis and Burleigh 2009; Amborella Genome Project 2013). Gene duplication provides new substrate for mutation and selection to act upon. In most cases, a new gene duplicate evolves neutrally, stochastically accumulating loss of function mutations (pseudogenzation). However, a fraction of duplicates might be retained when 1) gene duplication endows organisms with mutational robustness as a result of functional redundancy (Gu et al. 2003), 2) selection for increased gene dosage occurs (Conant and Wolfe 2008), or 3) accompanying acquisition of novel or specialized functions. From a population genetic perspective, relaxed purifying selection and positive Darwinian selection may contribute to functional specialization of gene duplicates (Moore and Purugganan 2003; Zhang 2003; Conant and Wolfe 2008). Positive selection (PS) promotes the fixation of mutations with advantageous fitness effects, while relaxed purifying selection can tolerate the fixation of specific mutations that may also contribute to the acquisition of new or specialized functions.

In this study, we classified genes from the *U. gibba*, *Mimulus guttatus* (*Mimulus*), *Solanum lycopersicum* (tomato), *Arabidopsis thaliana* (*Arabidopsis*), and *Vitis vinifera* (grape) genomes by clustering orthologous and in-paralogous genes from these five species into orthogroups, which we take to be equivalent to gene families. Gene families were classified as expanded/contracted in specific lineages or stable in size by means of the maximum likelihood (ML) framework provided by Badirate (Librado et al. 2012). We found that the orthogroups significantly expanded in the *U. gibba* lineage may have adaptive significance for the evolution of the species’ unique phenotypic features. Moreover, to identify signatures of adaptive functional specialization at the protein-coding sequence level, we performed a preliminary genome-wide scan for PS on the data set of co-orthologous groups that included representatives from the five species, and examined in further detail seven of the genes showing signatures of PS in the *U. gibba* lineage. Our results provide key genes and gene families that may be interesting targets for further experimental confirmation of their functional and adaptive roles in *U. gibba*’s unique lifestyle and highly specialized body plan.

## Materials and Methods

### Functional Annotation of Five Plant Eudicot Genomes and Statistical Analysis of Gene Ontology Term Enrichment/Depletion

All gene models predicted in the fully sequenced genomes of *Arabidopsis*, grape, *U. gibba*, *Mimulus,* and tomato were downloaded from CoGe (http://genomevolution.org/CoGe/) and functionally annotated by assigning their associated generic gene ontology (GO) terms and enzyme codes (EC) through the BlAST2GO program (Conesa et al. 2005), and integrating information about the occurrence of INTERPRO functional domains identified by INTERPROSCAN (Zdobnov and Apweiler 2001) and KEGG EC biochemical pathways (Ogata et al. 1999). Annotations were further expanded using ANNEX (Myhre et al. 2006). The following settings were used: BLAST searches were conducted for each protein (BLASTX, nr database, HSP cutoff length 33, report 20 hits, maximum *E*-value 1 E-10), followed by mapping and annotation (*E*-value hit filter 1 E-10, annotation cutoff 55, GO weight 5, HSP-hit coverage cutoff 20). As a gene might have more than one function, it might be annotated with more than one GO functional category or EC code. A substantial fraction of genes per species was associated with at least one GO generic term, ranging from 60.61% to 82.30% for *U. gibba* and *Arabidopsis* genes, respectively, averaging 4.62–5.44 associated GO terms per functionally annotated gene (table 1). To obtain a broader overview of functional annotation, the resulting generic GO terms were mapped onto the corresponding Plant GO slim terms. The GO and GOslim annotations of the five genomes are available as supplementary files S1 and S2, Supplementary Material online, respectively. We performed significance analyses of GO term enrichment/depletion of subsets of genes versus all genes in a genome by means of Fisher’s exact tests. To control for multiple testing, the resulting *P*-values were corrected according to Benjamini and Hochberg (1995).

**Table 1:** Numbers of Genes and GO Functional Annotations for Five Eudicot Plant Genomes

	*Utricularia gibba*	*Arabidopsis*	Grape	*Mimulus*	Tomato
Total number of genes	28,494	27,204	26,346	27,501	34,727
Total number of annotated genes	17,270	22,390	19,090	21,435	22,651
Annotated genes (%)	60.61	82.30	72.46	77.94	65.23
Total number of generic GO terms	79,933	118,023	92,018	102,601	107,423
Average number of generic GO terms/gene	4.63	5.27	4.82	4.79	4.74
Total number of plant GO slim terms	83,160	121,977	95,293	106,069	111,193
Average number of plant GO slim terms/gene	4.82	5.45	4.99	4.95	4.91

### Gene Family Definition Using OrthoMCL

The complete proteomes encoded by the genomes of *Arabidopsis*, grape, *U. gibba*, *Mimulus,* and tomato were globally compared. The complete data set represents 144,274 protein-coding sequences. An all-against-all comparison was performed using BLASTP (1 × 10 − 10) followed by clustering with OrthoMCL v1.4 (Li et al. 2003) with default parameters. To avoid possible enrichment of transposable element in our analyses of lineage-specific expansions, we filtered out orthogroups containing sequences with significant similarity to them (*E*-values **<** 10^−^^5^, bit score ≥45 in BLASTX searches against RepBase v16.03 database [Jurka et al. 2005]). The final data set (supplementary file S3, Supplementary Material online) contained 17,324 multigene orthogroups and 93,865 proteins.

### Lineage-Specific Gene Family Size Changes

To analyze the evolution of gene families, we applied gain and death (GD) stochastic models as implemented in the BadiRate program (Librado et al. 2012). BadiRate provides the appropriate statistical framework for testing biologically relevant hypothesis on the input data, consisting of the species phylogenetic tree and the sizes of gene families in each extant species. To detect species-specific rates of gene GD, we compared the fit of different branch models to the data applying the same approach as in Denoeud et al. (2014).

### Functional/Selective Constraints in Protein-Coding Regions

Selection may differentially act on nonsynonymous (amino acid-changing; d_N_) substitutions compared with the putatively neutral synonymous (silent; d_S_) ones. Under the neutral model of evolution, synonymous and nonsynonymous substitutions accumulate at the same rate (ω = d_N_/d_S_ ∼ 1; Ohno 1970; Li et al. 1981; Nei 2005). Conversely, deviations from this pattern indicate the action of purifying selection (which purges deleterious mutations to conserve the protein structure and function; ω ≪ 1), or the action of PS promoting the fixation of nonsynonymous mutations with advantageous fitness effects (ω > 1). Estimation of ω therefore yields insights on the molecular evolutionary mechanisms of protein diversification and functional specialization.

We estimated ω values by means of the codeml program in the PAML v4.4 package (Yang 1997) on the basis of multiple alignments of orthologous codon sequences and a tree topology. To obtain final codon alignments, the coding sequences were aligned with PRANK (Löytynoja and Goldman 2005) on the GUIDANCE server (http://guidance.tau.ac.il/; Penn et al. 2010), and the default cutoff of 0.93 confident score was applied for the removal of unreliably aligned positions.

Two different classes of models were implemented in the codeml program: “branch-specific” (which permit a different ω ratio in *U. gibba*; Yang 1998) and “branch-site” models (which also accounting for uneven selective pressures along sequences; Yang and Nielsen 2002). By comparing the fit to the data of two nested models via a likelihood ratio test, we tested for: 1) asymmetric sequence evolution (one-ratio model 0 versus the branch-specific model described above); 2) heterogeneous selective pressures across sites (ω ratio constant among branches but variable among sites versus the clade model described in Bielawski and Yang (2004)); and 3) PS (ω fixed at 1 at the *U. gibba* branch versus a model featuring an extra class of sites with ω > 1; Yang et al. 2005).

In a first approach, we used the original, entire set of 8,676 shared orthogroups containing homologs from all five species. Prior to the analyses, we discarded 1) sequences whose alignments spanned less than 150 nt or showed an identity score <30% (Li et al. 2001) (1,659 orthogroups), as too divergent and, 2) sequences containing potential errors leading to in-frame codon stops (169 orthogroups). This resulted in a set of 6,848 shared orthogroups well conserved among all five species. The entire process was automated using a PERL pipeline adapted from Carretero-Paulet and Fares (2012).

We further confirmed a subset of seven selected *U. gibba* genes detected as under PS in our previous analysis. For this purpose, we performed additional analyses of PS by sampling orthologs from a greater number of species (supplementary table S7, Supplementary Material online) and their corresponding tree topologies as returned from the NCBI taxonomy tree tool (http://www.ncbi.nlm.nih.gov/Taxonomy/CommonTree/wwwcmt.cgi). The orthology relationships of the sequences sampled were first assessed using COGE/GEvo (http://genomevolution.org/CoGe/GEvo.pl) to ascertain strong evidence for synteny, which was considered confirmation of (co-)orthology. In some cases, codon sequences were repredicted from the corresponding genomic sequences through comparisons with their orthologous proteins using GENEWISE (Birney and Durbin 2000).

### 3D Protein Structure Modeling

Protein structure models were generated using the SWISS-MODEL workspace (Arnold et al. 2006) on alignment mode and specific structure template selection. The structures of the *U. gibba* orthologs to AXR1, IGS, TAR2, SOL1, DEG9, and DEG10 were modeled using the protein structures with PDB identifiers 2nvuA (2.80 Å) (Huang et al. 2007), 3tsmA (2.15 Å), 3bwnB (2.25 Å) (Tao et al. 2008), 1h8lA (2.60 Å) (Aloy et al. 2001), and 4flnB (2.80 Å) (Sun et al. 2012), respectively, as templates. Protein 3D-structural models were displayed and edited using VMD 1.9.1 (Humphrey et al. 1996).

## Results and Discussion

### Analysis of Gene Family Turnover in *U. gibba*

**Fig. 1.—:**
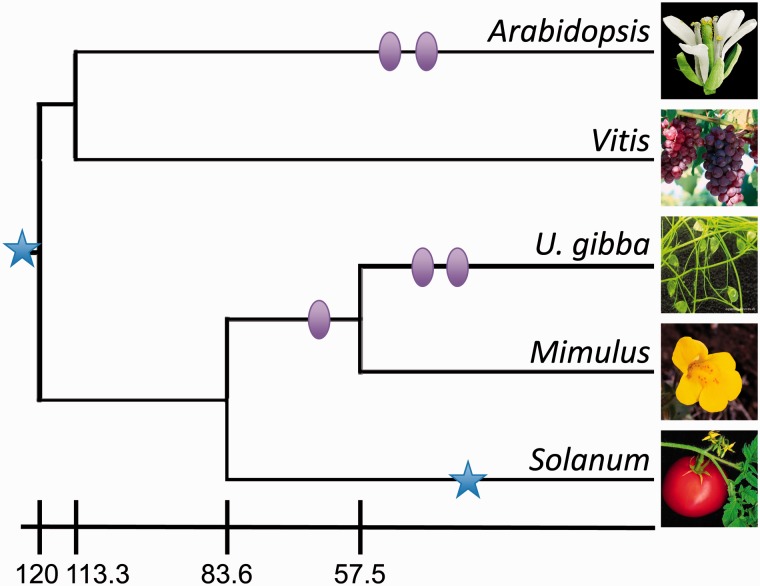
Phylogenetic tree depicting the taxonomic relationships among the five plant species with fully sequenced genomes examined in this study. Branch lengths reflect evolutionary time (in millions of years). For the timing of these events, we used estimates from TimeTree (Hedges et al. 2006), which are shown next to the scale bar. The history of WGDs is mapped onto the tree, with circles and stars representing WGDs and triplications, respectively. The positions of these events are not meant to reflect their timing of occurrence.

Genes in the genomes of *U. gibba*, the asterids *Mimulus* and tomato, and the rosids *Arabidopsis* and grape, which represent highly diverse core eudicot lineages with different histories of WGD (fig. 1 and table 1) were classified into groups of orthologous genes (orthogroups) using OrthoMCL. We used the resulting set of 17,324 orthogroups to examine the relationship between the *U. gibba* genome's compactness and its rates of gene turnover, while accounting for their potential adaptive value for the *U. gibba* evolution. We evaluated the fits of different branch models of gene turnover to each orthogroup using the statistical framework provided by BadiRate (Librado et al. 2012). According to the ML best-fit branch model of gene turnover, each orthogroup was classified as 1) lineage-specific expanded or contracted, or 2) stable in size across species (supplementary table S1, Supplementary Material online). Six hundred twenty-six orthogroups (comprising 1,754 *U. gibba* genes) were considered significantly expanded in the *U. gibba* lineage (supplementary tables S2 and S3, Supplementary Material online), whereas 628 orthogroups (comprising only eight *U. gibba* genes) were classified as contracted, having in most cases lost all of their *U. gibba* orthologs (supplementary tables S4 and S5, Supplementary Material online). Six thousand one hundred seventy orthogroups (comprising 6,188 *U. gibba* genes) corresponded to orthogroups showing constant sizes across all five genomes.

Although some genes could be incorrectly annotated or biologically not meaningful, GO annotations represent the best approach developed so far to gain further insights into the potential adaptive value of the *U. gibba*-specific expansions. In particular, we performed a GO enrichment analysis (GO terms in genes belonging to expanded orthogroups versus all GO terms in the *U. gibba* genome), partitioned by either generic GO terms or Plant GO slim terms (table 1 and supplementary tables S2 and S3, Supplementary Material online). Seven GO slim terms (out of a total of 104, including two significant after correction for multiple testing) and 94 generic GO terms (out of a total of 4,330, including 21 significant after correction for multiple testing) were found to be significantly and specifically enriched among *U. gibba*-expanded orthogroups (table 2). Given the compact genome size of *U. gibba*, the expansion of specific gene families may reflect the emergence of key *U. gibba* adaptations, such as physiological and morphological phenotypic plasticity (assuming that our GO annotations are essentially correct, and some degree of uncertainty in specific GO terms with a low number of genes). After careful examination of the GO terms found as enriched in expanded orthogroups, we operationally grouped them into three main biological functions that are explored in more detail below. These biological functions are: 1) trap physiology, 2) key plant morphogenetic/developmental pathways, and 3) response to environmental stimuli, including adaptations to life in aquatic environments.

**Table 2:** Generic and Plant GO Slim Terms Enriched among Gene Families Expanded in *Utricularia gibba*

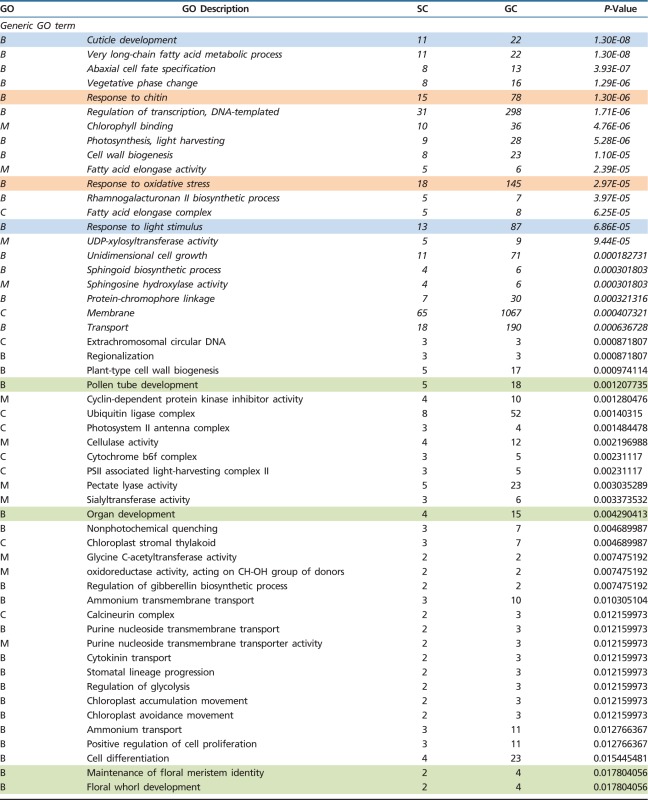
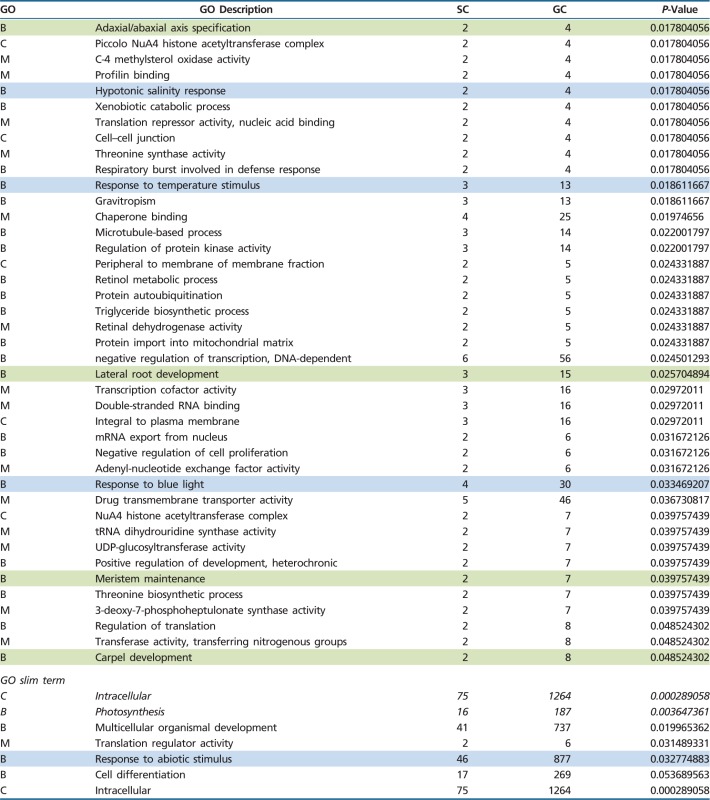

Note.—B, M and C indicate biological process, molecular function and cellular component GO classes, respectively. Sample counts and genome counts indicate the total numbers of genes annotated with particular GO terms among expanded families (of which there are 1,754 genes total) versus the entire *U. gibba* genome (which contains a total of 28,494 genes). *P*-values resulting from Fisher exact tests are shown (in italic, those significant after Benjamini–Hochberg correction). Selected GO terms generically related to 1) trap physiology, 2) plant morphogenetic/developmental pathways, and 3) response to environmental stimuli and adaptations to life in aquatic environments are colored orange, green, and blue, respectively.

### The Putative Role of Gene Family Expansion in the Evolution of Trap Physiology in *U. gibba*

To understand the molecular evolutionary basis of the *U. gibba* carnivorous syndrome, we explored functional enrichments among expanded orthogroups that may be related to trap physiology. Since *U. gibba* can acquire nutrients from prey captured by its bladder traps, the digestion of these organisms is a critical function. Various enzymes, such as hydrolases, peptidases, phosphatases, and chitinases have been proposed to be relevant to prey digestion among different carnivorous plant lineages (Sirová et al. 2003; Muller et al. 2004, 2006; Plachno et al. 2006; Hatano and Hamada 2008; Rottloff et al. 2011; Renner and Specht 2013), which independently evolved across angiosperm phylogeny (Ellison and Gotelli 2009).

We found that the generic GO term “response to chitin” was significantly enriched after correction among gene families expanded in the *U. gibba* lineage (table 2 and supplementary table S2, Supplementary Material online). Chitin is the main component of the cell walls of fungi and also the exoskeleton of arthropods and nematodes, and is a well-known elicitor of plant defense responses (Shibuya and Minami 2001; Kaku et al. 2006). Subclass I chitinases have been proposed to play a major role in pathogenic response in plants (Salzer et al. 2000), and have also been suggested to serve plant carnivory in the order Caryophyllales (Renner and Specht 2012). Although no GO term specifically related to “chitinase” was enriched among the *U. gibba* gene family expansions, the enrichment of the response to chitin GO term may reflect adaptation to chitin detection and activation of relevant biochemical pathways involved in prey digestion, nutrient absorption, and/or in defense against pathogenic fungi.

The production of enzymes for prey digestion and nutrient acquisition, together with the lower photosynthetic capacity of trap biomass compared with leaf-like structures in *Utricularia*, implies a high energetic cost (Adamec 2006). Indeed, a delicate balance between energetic costs and benefits of prey digestion and nutrient acquisition has been proposed for bladderworts (Ellison and Gotelli 2001; Laakkonen et al. 2006). Previous research has suggested that respiratory rates are high in *Utricularia* traps, and that this could result from adaptive protein changes in cytochrome *c* oxidase (COX), a primary enzyme of the electron transport chain (Laakkonen et al. 2006). It is well known that high charge differences between the mitochondrial lumen and intermembrane space can lead to leakage of electrons and the production of reactive oxygen species (ROS; Turrens 2003; Albert et al. 2010). Interestingly, the generic GO term “response to oxidative stress” was found to be specifically enriched (significant after correction) among the *U. gibba*-expanded gene families (table 2 and supplementary table S2, Supplementary Material online). The potential adaptive value of this expansion is consistent with previous transcriptomic studies in *U. gibba* that support the hypothesis that COX-derived ROS may account for both increased nucleotide substitution rates and genome-size dynamism following DNA double-strand break repair (Ibarra-Laclette et al. 2011).

We also found the generic GO term “respiratory burst involved in defense response” specifically enriched among *U. gibba*-expanded gene families. This GO term exclusively annotates to ORTHOMCL15238, an orthogroup entirely composed of three *U. gibba* PUB23-like E3 ubiquitin-protein ligases. *PUB23*, similarly to other *PUB* family genes in *Arabidopsis*, likely regulates drought and plant pathogen immunity signaling pathways (Cho et al. 2008). Interestingly, the enhanced respiratory burst in *PUB* mutants might be triggered by chitin (Trujillo et al. 2008). Respiratory burst in response to pathogens can rapidly release ROS such as superoxide and hydrogen peroxide, which play roles in both direct killing of pathogens and induction of defense gene expression (Bolwell 1999). Because plant carnivory may have evolved from defense mechanisms against herbivores and pathogens (Mithofer 2011; Renner and Specht 2013), the possible pathogen defensive role of these *PUB23*-like genes may have been recruited to *U. gibba*'s carnivorous syndrome, perhaps in response to prey capture. The expansion of the *PUB23*-like gene family in *U. gibba* is also consistent with increased ROS levels in traps versus vegetative structures.

### The Putative Role of Gene Family Expansion in the Evolution of *U. gibba’s* Specialized Body Plan

*Utricularia gibba* shows a unique and highly specialized body plan, including absence of roots and presence of fibrous floating networks of photosynthetic structures and trapping bladders. Interestingly, the generic GO term “lateral root development” was enriched among the *U. gibba*-expanded families (table 1 and supplementary table S2, Supplementary Material online). In total, three *U. gibba* genes belong to this functional category; two of them constitute ORTHOMCL18706 and another belongs to ORTHOMCL18829.

The *U. gibba* genes in ORTHOMCL18706 are likely homologous to the five highly conserved *Arabidopsis* genes *BREVIS RADIX* (*BRX*) and *BREVIS RADIX-like 1-4* (Mouchel et al. 2004; Briggs et al. 2006); *BRX* regulates root meristematic growth, possibly by controlling auxin response factor activity. ORTHOMCL18829 is composed of two *U. gibba* members encoding WRKY transcription factors, with only one of them annotated with the term lateral root development. Interestingly, when we used the corresponding sequences as queries to perform BLAST searches on the *Arabidopsis* genome, the best retrieved hit was *AtWRKY75* (AT5G13080), which plays a regulatory role in root hair patterning (Rishmawi et al. 2014). These two examples of enrichment for lateral root function may reflect gene functional specialization by co-option of orthologous genes during *U. gibba* evolution. The fact that *U. gibba* has no roots could suggest a heterotopic transfer of function of these genes to other organs, possibly ones bearing trichomes, which are developmentally related to root hairs. Indeed, bladder trichomes are the sites of nutrient absorption in *U. gibba*, so it is tempting to speculate that some ancestral “root-specific” functions may have been transferred to traps. Indeed, both genes in ORTHOMCL18829 are preferentially expressed in bladders (Ibarra-Laclette et al. 2011). A potentially similar instance of evolutionary co-option may come from research on moss orthologs of the *Arabidopsis* bHLH transcription factors RHD6 and AtRSL1, reported to control root hair development. The corresponding orthologs in moss, PpRSL1 and PpRSL2, control the development of rhizoids, which are nonhomologous organs with a rooting function (Menand et al. 2007).

Furthermore, two genes annotated with the term “meristem maintenance,” likely homologous to *Arabidopsis HOMEOBOX GENE1* (*ATH1*, AT4G32980), were also found as enriched in expanded families. *ATH1* encodes a homeobox transcription factor involved in different photomorphogenic processes (Quaedvlieg et al. 1995), including modulation of growth at the interface between the stem, meristem, and organ primordia (Gómez-Mena and Sablowski 2008), and consequently, inflorescence architecture (Li et al. 2012).

### The Putative Role of Gene Family Expansion in the Adaptation to Life in Aquatic Environments

*Utricularia gibba* lives in various habitats, including as a suspended aquatic or as a creeping plant in marshlands. It has been reported that the morphology of *Utricularia* species is plastic, and highly affected by environmental factors such as temperature, light, and water level (Taylor 1989; Reut and Fineran 2000). Accordingly, many GO term enrichments among *U. gibba*-expanded orthogroups are associated with response to environmental stimuli, including “response to abiotic stimulus,” several related to light (“response to light stimulus”—significant after correction, “response to blue light,” and “nonphotochemical quenching”), temperature (“response to temperature stimulus”), or salinity (“hypotonic salinity response”). The enrichments of these functional categories are consistent with key roles for various environmental stimuli on *U. gibba* development and physiology: *U. gibba* can live in very dynamic habitats that frequently transition among different environmental conditions. The rapid and efficient control of various metabolic and developmental responses to changing environmental constraints may therefore be critical to *U. gibba* survival.

For example, under typical environmental conditions, *U. gibba* produces normal, open bilateral flowers. However, under low light conditions, the lips are closed and the corollas do not open. Likewise, in deeper water or a submerged environment, the flowers lose corollas altogether and bear only closed calyces (Chao 2003). Both types of cleistogamous flowers are fertile and undergo self-fertilization. It is tempting to speculate that the GO term enrichment related to response to environmental stimuli may reflect the importance of fine-tuned sensing of environmental factors participating in proper switching among flower-type developmental pathways.

In keeping with these unique flowering features, we discovered several enriched GO terms related to floral (meristem) development, such as “maintenance of floral meristem identity,” “meristem maintenance,” “floral whorl development,” “organ development,” “pollen tube development,” “adaxial/abaxial axis specification,” and “carpel development” (table 2 and supplementary table S2, Supplementary Material online). Among the genes annotated with the term maintenance of floral meristem identity, two clustered within orthogroup ORTHOMCL11149, an orthogroup comprising five *U. gibba* members likely homologous to *Arabidopsis SHORT VEGETATIVE PHASE* (*SVP,* AT2G22540). *SVP* encodes a MADS-box transcription factor that acts as a central regulator of flowering time (Hartmann et al. 2000). Although available expression data show only low to null expression in flowers (Ibarra-Laclette et al. 2011), the *U. gibba*-specific expansion of this family may be related to the critical function of *SVP-*like genes in the species’ flowering phenology.

Another highly significant enriched functional category (after correction) that may relate to the aquatic habit was “cuticle development.” Eleven of the 22 genes annotated with this term belong to the *U. gibba*-expanded orthogroup ORTHOMCL309, composed of 11 *U. gibba* members and 0–4 representatives from the other species. All of these genes are annotated as coding for 3-ketoacyl-CoA synthase enzymatic activity, involved in the biosynthesis of very long-chain fatty acids. The two *Arabidopsis* orthologs belonging to this orthogroup, AT1G25450 (*CER6*) and AT1G68530 (*CUT1*), catalyze the first step of the biosynthetic pathway for the layer of wax secreted onto the aerial surfaces of all land plants. This layer of wax protects plants from desiccation and other stresses, and allows survival in terrestrial environments (Millar et al. 1999; Fiebig et al. 2000). This hydrophobic cuticle layer evolved in land plants during the transition from an aquatic to a terrestrial environment (Go et al. 2014). The preferential retention and potential further functional diversification of duplicated genes from this family may reflect the metabolic flexibility required for waxy cuticle production in order to properly deal with the changing environments characteristic of *U. gibba*’s lifestyle. Accordingly, genes belonging to ORTHOMCL309 show broad and diverse expression patterns (Ibarra-Laclette et al. 2011).

### Putative Roles of Adaptive Functional Specialization in *U. gibba* Phenotypic Diversification: A Case Study of Seven Candidate Genes

To further grasp the molecular evolutionary signatures underlying the evolution of *U. gibba*’s specialized body plan and unique lifestyle, we performed a preliminary genome-wide analysis of selection on the protein-coding regions of the 6,848 orthogroups containing homologs from all five species, and that exceeded the quality criteria filters (see Materials and Methods; supplementary table S6, Supplementary Material online). Different codon-substitution evolutionary models assessing for asymmetric evolution, divergent selective pressures or PS specifically occurring in the *U. gibba* ortholog were implemented in PAML (Yang 1997, 2007). From these preliminary results, we selected seven candidate genes that showed the molecular hallmarks of PS in the *U. gibba* lineage. These seven cases were subjected to manual curation and further examination for PS using a greater sampling of orthologous sequences (table 3).

**Table 3:** Summary of Genes Identified as under PS in *U. gibba*

Gene	*Arabidopsis* Ortholog	Function in *Arabidopsis*
*Scf00011.g1809.t1*	*AXR1*	A subunit of the RUB1-activating enzyme that regulates the protein degradation activity of Skp1-Cullin-Fbox complexes, which primarily, but not exclusively, affect auxin responses
*Scf00146.g10482.t1*	*UMAMIT41*	A nodulin *MtN21*-like transporter family protein that has a possible role in response to auxin in adventitious root formation
*Scf00260.g14017.t1*	*IGS*	Catalyzes the fourth step of the tryptophan biosynthesis pathway, and is as such responsible for the synthesis of indole-3-glyceralphosphate, the intermediate serving as a branchpoint to the Trp-independent pathway for auxin biosynthesis
*Scf00083.g7570.t1*	*TAR2*	Involved in the indole-3-pyruvic acid (IPyA) pathway, one of the Trp-dependent pathways for auxin biosynthesis. Together with *TAA1,* affects root meristem maintenance and differential growth in apical hooks
*Scf00505.g18737.t1*	*SOL1*	A suppressor of root-specific overexpression of *CLE19*. The *sol1* mutant partially suppresses the loss of root meristem maintenance and short root phenotype caused by *CLE19* overexpression
*Scf00003.g611.t1*	*DEG9*	The only Deg protease member located in the nucleus. Hypothesized to have functions involving ribosome-related transcription and modification.
*Scf00324.g15492.t1*	*DEG10*	Highly induced after treatment with inhibitors of the mitochondrial electron transport chain, which suggests a role of DEG10 in protein quality control via degradation of damaged mitochondrial proteins

First, we focused on four genes involved in several critical steps of auxin metabolism, signaling of primary root abortion, and vegetative meristem growth, as these may have played a major role in the evolution of *U. gibba*’s specialized body plan. For example, *Scf00011.g1809.t1* is orthologous to *Arabidopsis AXR1* (At1G05180; supplementary fig. S1, Supplementary Material online), a gene regulating the protein degradation activity of Skp1-Cullin-Fbox complexes, which primarily affect auxin responses (Leyser et al. 1993; del Pozo et al. 2002). Similarly, *Scf00146.g10482.t1* is orthologous to *Arabidopsis UMAMIT41* (At3G28050; supplementary fig. S2, Supplementary Material online), which is a nodulin *MtN21*-like transporter family protein that may respond to IAA in adventitious root formation (Busov et al. 2004). Beside these two auxin/IAA signaling related genes, we also selected *Scf00260.g14017.t1*, an ortholog of *indole-3-glycerol phosphate synthase* (*IGS*) (AT2G04400; supplementary fig. S3, Supplementary Material online), partly responsible for the synthesis of indole-3-glyceralphosphate, which is the intermediate serving as a branch point to the Trp-independent pathway for auxin synthesis (Tzin and Galili 2010). Furthermore, another gene identified as subjected to PS in *U. gibba* was *Scf00083.g7570.t1*, an ortholog of *tryptophan aminotransferase related 2* (*TAR2*; supplementary fig. S4, Supplementary Material online), involved in the indole-3-pyruvic acid (IPyA) pathway, one of the Trp-dependent pathways for IAA biosynthesis. By generating auxin gradients among different parts of plant tissue, *TAR2* and *TAA1* affect root meristem maintenance and differential growth in apical hooks (Stepanova et al. 2008). Therefore, our analysis suggests an evolutionary fine-tuning of genes involved in inhibiting primary root elongation, which can stimulate the initiation of lateral and adventitious roots (Vanneste and Friml 2009).

Another gene found to be evolving under PS in *U. gibba* was *Scf00505.g18737.t1*, an ortholog of *Arabidopsis SOL1* **(**AT1G71696; supplementary fig. S5, Supplementary Material online). *SOL1* was isolated as a suppressor of root-specific overexpression of *CLE19*, a *CLAVATA3-like* gene (Casamitjana-Martınez et al. 2003). Because the *sol1* mutant partially suppresses the loss of root meristem maintenance and short root phenotype caused by *CLE19* overexpression, the signature of PS found in the *SOL1* ortholog might reflect a similar function related to arrested root differentiation.

Finally, two additional *U. gibba* genes examined were *Scf00003.g611.t1* and *Scf00324.g15492.t1*, orthologs of *Arabidopsis DEG9* (AT5G40200) and *DEG10* (AT5G36950), respectively (supplementary figs. S6 and S7, Supplementary Material online). According to the MEROPS database classification (http://merops.sanger.ac.uk/), *DEG9* and *DEG10* belong to the peptidase family S1 (chymotrypsin family), which is widely distributed throughout all kingdoms of life. In animals, these peptidases play many different roles, including intestinal digestion and IgA-mediated immune responses (Lister et al. 2004). The functions of these peptidases in plants are not well known. *Arabidopsis* DEG9 was found as the only Deg protease member to be located in the nucleus, and was hypothesized to have functions involving ribosome-related transcription and modification. *DEG10* was reported to be highly induced after treatment of *Arabidopsis* plants with inhibitors of the mitochondrial electron transport chain, which suggested a role of DEG10 in protein quality control via degradation of damaged mitochondrial proteins (Lister et al. 2004). Apart from the tempting hypothesis that they may also play a role in prey digestion, these Deg proteases may provide essential protection against damage to cellular proteins, for example, as incurred by ROS.

**Fig. 2.—:**
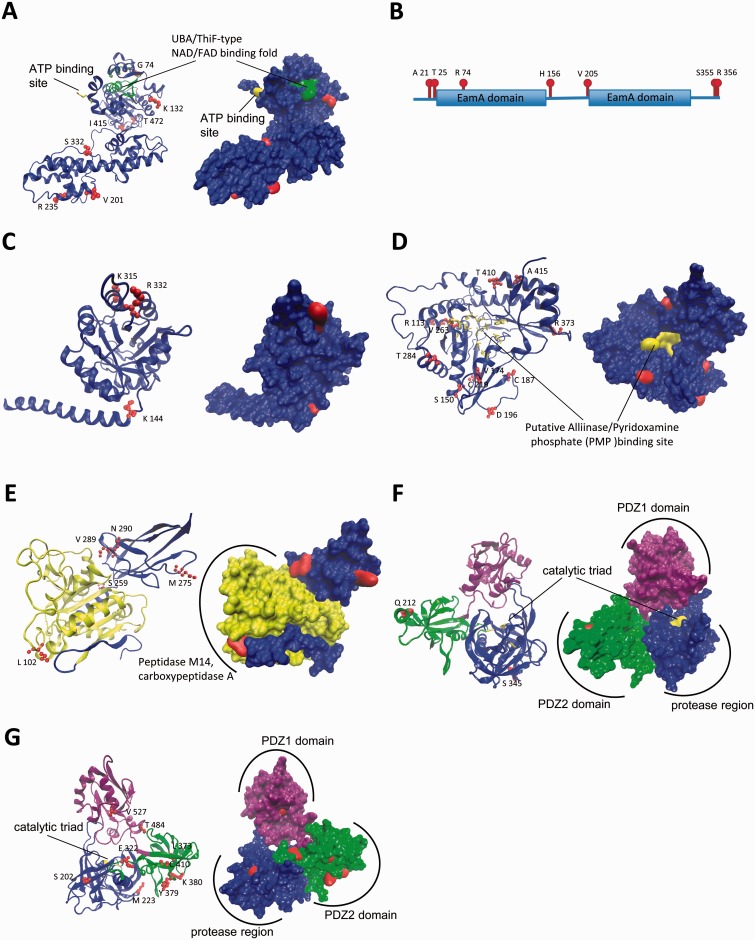
Protein 3D-structure models and architecture of functional domains of seven *Utricularia gibba* genes. (*A* and *C–G*) Cartoon backbones highlighting secondary structures (left side of the panel) and molecular surface representations (right side of the panel) for *U. gibba* AXR1, IGS, TAR2, SOL1, DEG9, and DEG10, respectively. The 3D-structural models were obtained using SWISS MODEL. (*B*) Architecture of protein functional domains of *U. gibba* UMAMIT41 as retrieved from INTERPROSCAN. Putative PS residues are shown in red. Functionally relevant residues or protein domains are shown in different colors.

Many of the PS amino acid sites found in *U. gibba* orthologous proteins occurred in positions well conserved in the remaining sequences included in alignments, and some involved radical changes in the physicochemical properties of the amino acids (fig. 2 and supplementary figs. S1–S7, Supplementary Material online). To gain further insights into the putative functional roles of the amino acid changes fixed by PS, we mapped them onto 3D-structural models when available, or the protein functional domains as predicted by INTERPROSCAN (Zdobnov and Apweiler 2001). In each case, functionally significant residues were identified from the literature or predicted by Evolutionary Trace (Lichtarge et al. 1996; fig. 2). Interestingly, some of the PS amino acid site changes were located within functional domains or residues, suggestive of their possible biological significance. For example, two of the residues (174 V and 187 C) inferred to be subjected to PS in the *U. gibba* TAR2 ortholog were within the protein's surface patch (Tao et al. 2008; fig. 2*D*). In addition, in the *U. gibba* DEG9 ortholog, one PS residue (212 Q) was located in the PDZ2 domain, whereas another (345 S) occurred in the protease domain (fig. 2*F*). In *U. gibba* DEG10 orthologs, five PS residues (322 E, 373 L, 379 Y, 380 K, and 410 C) were in the PDZ2 domain, whereas two additional PS residues (202 S and 223 M) lay in the protease region (fig. 2*G*). Since the PDZ2 domain mediates hexamer formation and locks the protease into the resting state (Sun et al. 2012), its PS amino acid changes may modify their possible coordination in specific protein functions.

## Conclusions

We found examples of both lineage-specific gene family expansions and putative adaptive functional specialization of *U. gibba* orthologs that could have contributed to *U. gibba* evolution. Our genome-wide analyses of gene family expansions and protein-coding gene evolution provide useful information to explore possible mechanisms underlying both morphological and physiological specializations of *U. gibba*. Our results further suggest interesting target genes and gene families for future experimental confirmation to validate their possible adaptive functional roles in *U. gibba*’s highly specialized body plan and unique carnivorous lifestyle.

## Supplementary Material

Supplementary tables S1–S7, figures S1–S7, and files S1–S3 are available at *Genome Biology and Evolution* online (http://www.gbe.oxfordjournals.org)

Supplementary Data
