# Supplementary material for: Genome-Wide Analysis of Adaptive Molecular Evolution in the Carnivorous Plant Utricularia gibba
Source: Genome Biol Evol. 2015 Jan 9;7(2):444–56. doi: 10.1093/gbe/evu288 (PMC4350169; doi:10.1093/gbe/evu288)
Supplement: Supplementary Data [file supp_7_2_444__index.html]

Genome-wide analysis of adaptive molecular evolution in the carnivorous plant Utricularia gibba — Genome-Wide Analysis of Adaptive Molecular Evolution in the Carnivorous Plant Utricularia gibba — Supplementary Data 

# Genome-Wide Analysis of Adaptive Molecular Evolution in the Carnivorous Plant *Utricularia gibba*

## Supplementary Data

files

**Files in this Data Supplement:**

- Supplementary Data - zip file
