# Supplementary material for: Genome-Wide Analysis of Adaptive Molecular Evolution in the Carnivorous Plant Utricularia gibba
Source: Genome Biol Evol. 2015 Jan 9;7(2):444–56. doi: 10.1093/gbe/evu288 (PMC4350169; doi:10.1093/gbe/evu288)
Supplement: Supplementary Data [file supp_evu288_suppl_data.zip › SupplFigures UtriculariaVGBE_THC.pptx]

## Slide 1
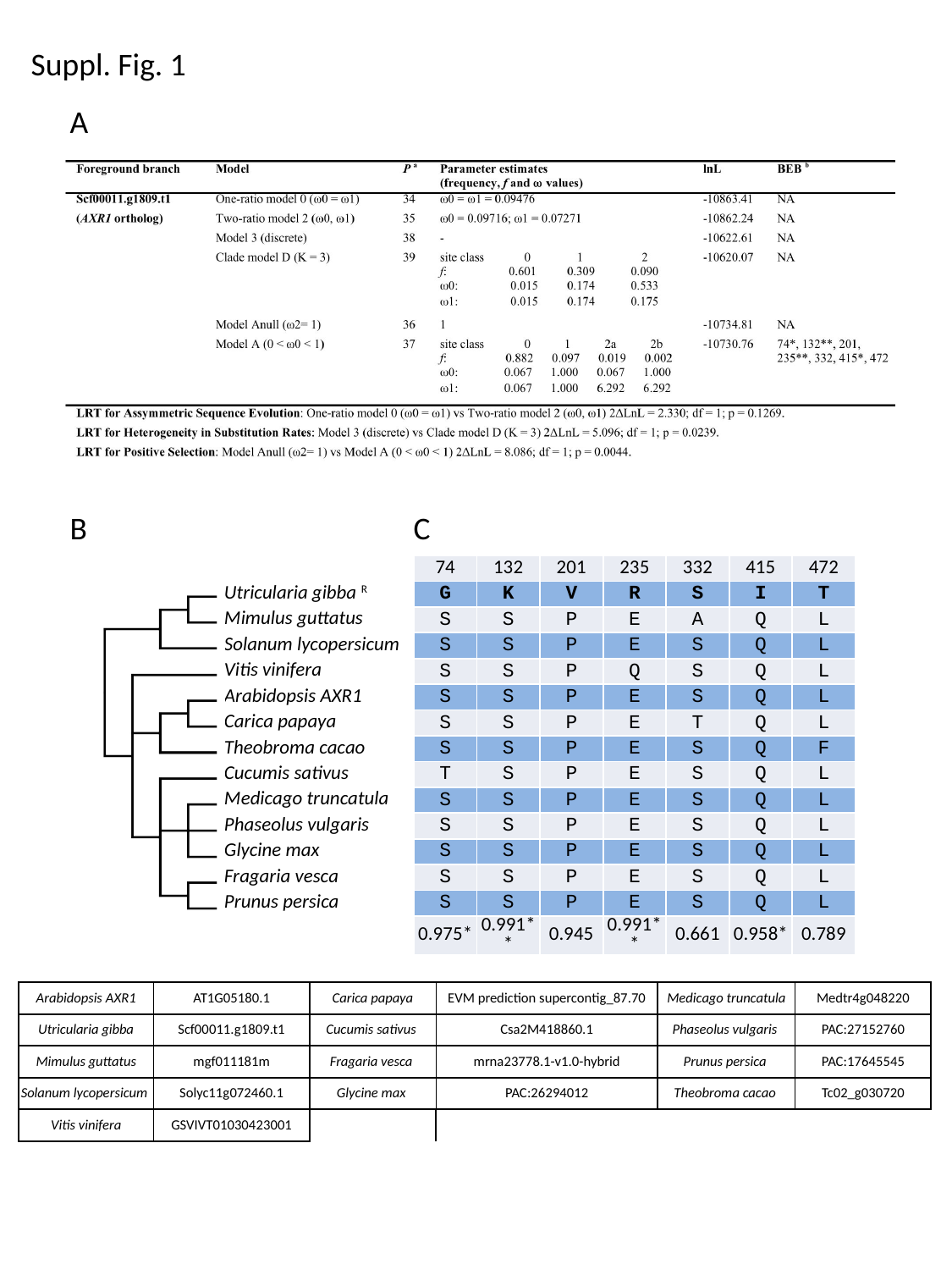

Suppl. Fig. 1
A
B
C
| | 74 | 132 | 201 | 235 | 332 | 415 | 472 |
| --- | --- | --- | --- | --- | --- | --- | --- |
| Utricularia gibba R | G | K | V | R | S | I | T |
| Mimulus guttatus | S | S | P | E | A | Q | L |
| Solanum lycopersicum | S | S | P | E | S | Q | L |
| Vitis vinifera | S | S | P | Q | S | Q | L |
| Arabidopsis AXR1 | S | S | P | E | S | Q | L |
| Carica papaya | S | S | P | E | T | Q | L |
| Theobroma cacao | S | S | P | E | S | Q | F |
| Cucumis sativus | T | S | P | E | S | Q | L |
| Medicago truncatula | S | S | P | E | S | Q | L |
| Phaseolus vulgaris | S | S | P | E | S | Q | L |
| Glycine max | S | S | P | E | S | Q | L |
| Fragaria vesca | S | S | P | E | S | Q | L |
| Prunus persica | S | S | P | E | S | Q | L |
| | 0.975\* | 0.991\*\* | 0.945 | 0.991\*\* | 0.661 | 0.958\* | 0.789 |
| Arabidopsis AXR1 | AT1G05180.1 | Carica papaya | EVM prediction supercontig\_87.70 | Medicago truncatula | Medtr4g048220 |
| --- | --- | --- | --- | --- | --- |
| Utricularia gibba | Scf00011.g1809.t1 | Cucumis sativus | Csa2M418860.1 | Phaseolus vulgaris | PAC:27152760 |
| Mimulus guttatus | mgf011181m | Fragaria vesca | mrna23778.1-v1.0-hybrid | Prunus persica | PAC:17645545 |
| Solanum lycopersicum | Solyc11g072460.1 | Glycine max | PAC:26294012 | Theobroma cacao | Tc02\_g030720 |
| Vitis vinifera | GSVIVT01030423001 | | | | |

## Slide 2
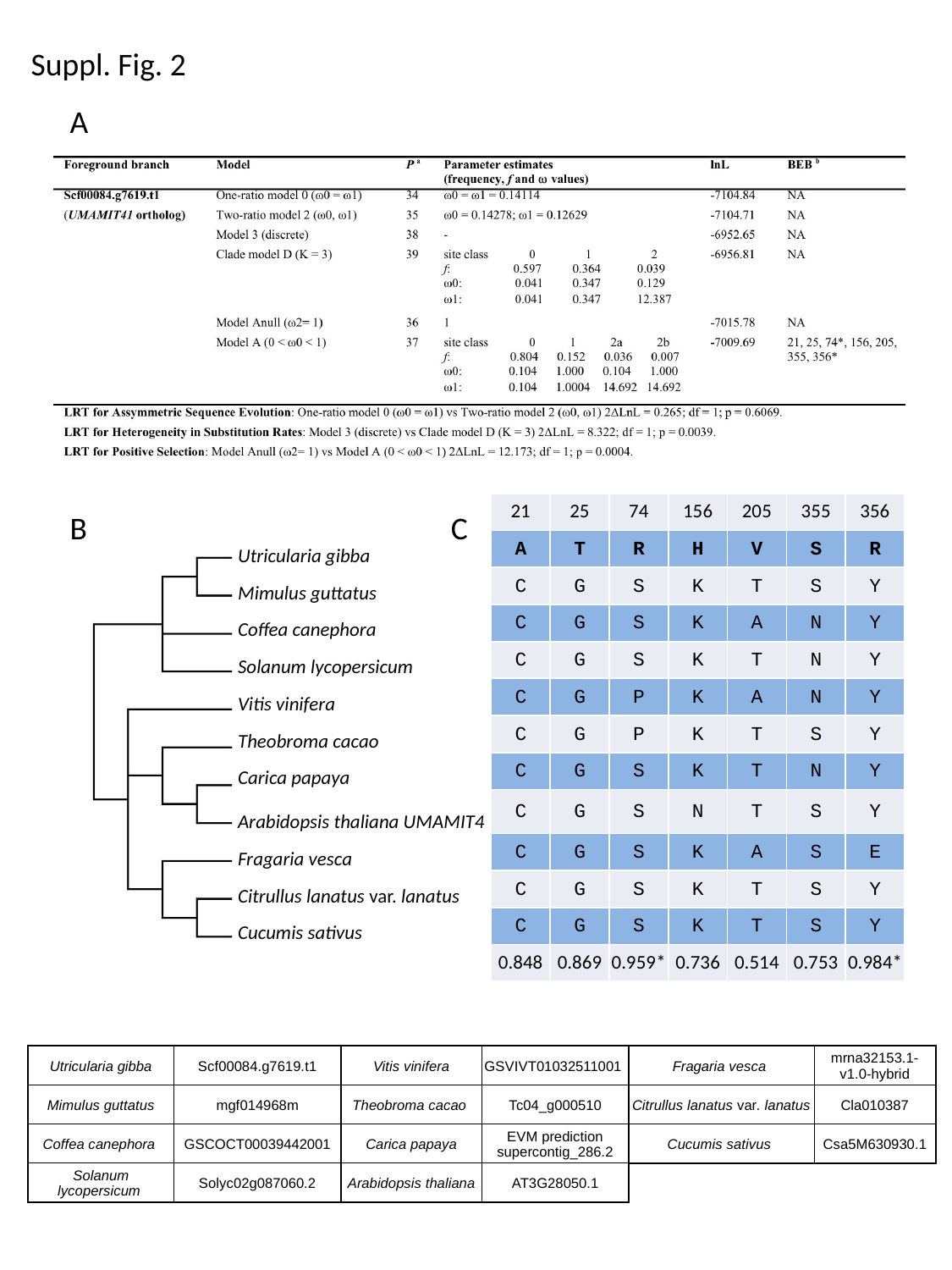

Suppl. Fig. 2
A
| | 21 | 25 | 74 | 156 | 205 | 355 | 356 |
| --- | --- | --- | --- | --- | --- | --- | --- |
| Utricularia gibba | A | T | R | H | V | S | R |
| Mimulus guttatus | C | G | S | K | T | S | Y |
| Coffea canephora | C | G | S | K | A | N | Y |
| Solanum lycopersicum | C | G | S | K | T | N | Y |
| Vitis vinifera | C | G | P | K | A | N | Y |
| Theobroma cacao | C | G | P | K | T | S | Y |
| Carica papaya | C | G | S | K | T | N | Y |
| Arabidopsis thaliana UMAMIT4 | C | G | S | N | T | S | Y |
| Fragaria vesca | C | G | S | K | A | S | E |
| Citrullus lanatus var. lanatus | C | G | S | K | T | S | Y |
| Cucumis sativus | C | G | S | K | T | S | Y |
| | 0.848 | 0.869 | 0.959\* | 0.736 | 0.514 | 0.753 | 0.984\* |
B
C
| Utricularia gibba | Scf00084.g7619.t1 | Vitis vinifera | GSVIVT01032511001 | Fragaria vesca | mrna32153.1-v1.0-hybrid |
| --- | --- | --- | --- | --- | --- |
| Mimulus guttatus | mgf014968m | Theobroma cacao | Tc04\_g000510 | Citrullus lanatus var. lanatus | Cla010387 |
| Coffea canephora | GSCOCT00039442001 | Carica papaya | EVM prediction supercontig\_286.2 | Cucumis sativus | Csa5M630930.1 |
| Solanum lycopersicum | Solyc02g087060.2 | Arabidopsis thaliana | AT3G28050.1 | | |

## Slide 3
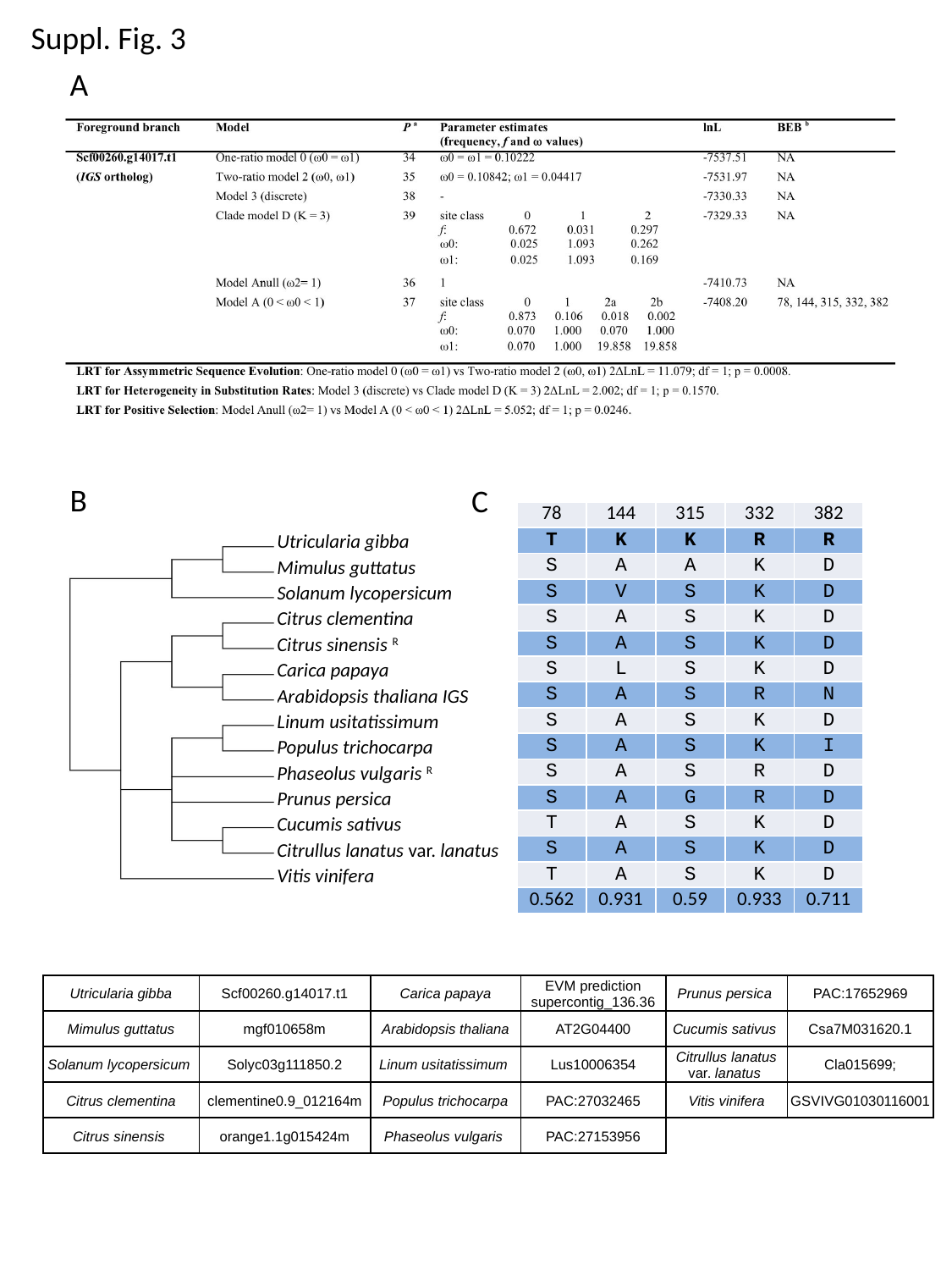

Suppl. Fig. 3
A
B
C
| | 78 | 144 | 315 | 332 | 382 |
| --- | --- | --- | --- | --- | --- |
| Utricularia gibba | T | K | K | R | R |
| Mimulus guttatus | S | A | A | K | D |
| Solanum lycopersicum | S | V | S | K | D |
| Citrus clementina | S | A | S | K | D |
| Citrus sinensis R | S | A | S | K | D |
| Carica papaya | S | L | S | K | D |
| Arabidopsis thaliana IGS | S | A | S | R | N |
| Linum usitatissimum | S | A | S | K | D |
| Populus trichocarpa | S | A | S | K | I |
| Phaseolus vulgaris R | S | A | S | R | D |
| Prunus persica | S | A | G | R | D |
| Cucumis sativus | T | A | S | K | D |
| Citrullus lanatus var. lanatus | S | A | S | K | D |
| Vitis vinifera | T | A | S | K | D |
| | 0.562 | 0.931 | 0.59 | 0.933 | 0.711 |
| Utricularia gibba | Scf00260.g14017.t1 | Carica papaya | EVM prediction supercontig\_136.36 | Prunus persica | PAC:17652969 |
| --- | --- | --- | --- | --- | --- |
| Mimulus guttatus | mgf010658m | Arabidopsis thaliana | AT2G04400 | Cucumis sativus | Csa7M031620.1 |
| Solanum lycopersicum | Solyc03g111850.2 | Linum usitatissimum | Lus10006354 | Citrullus lanatus var. lanatus | Cla015699; |
| Citrus clementina | clementine0.9\_012164m | Populus trichocarpa | PAC:27032465 | Vitis vinifera | GSVIVG01030116001 |
| Citrus sinensis | orange1.1g015424m | Phaseolus vulgaris | PAC:27153956 | | |

## Slide 4
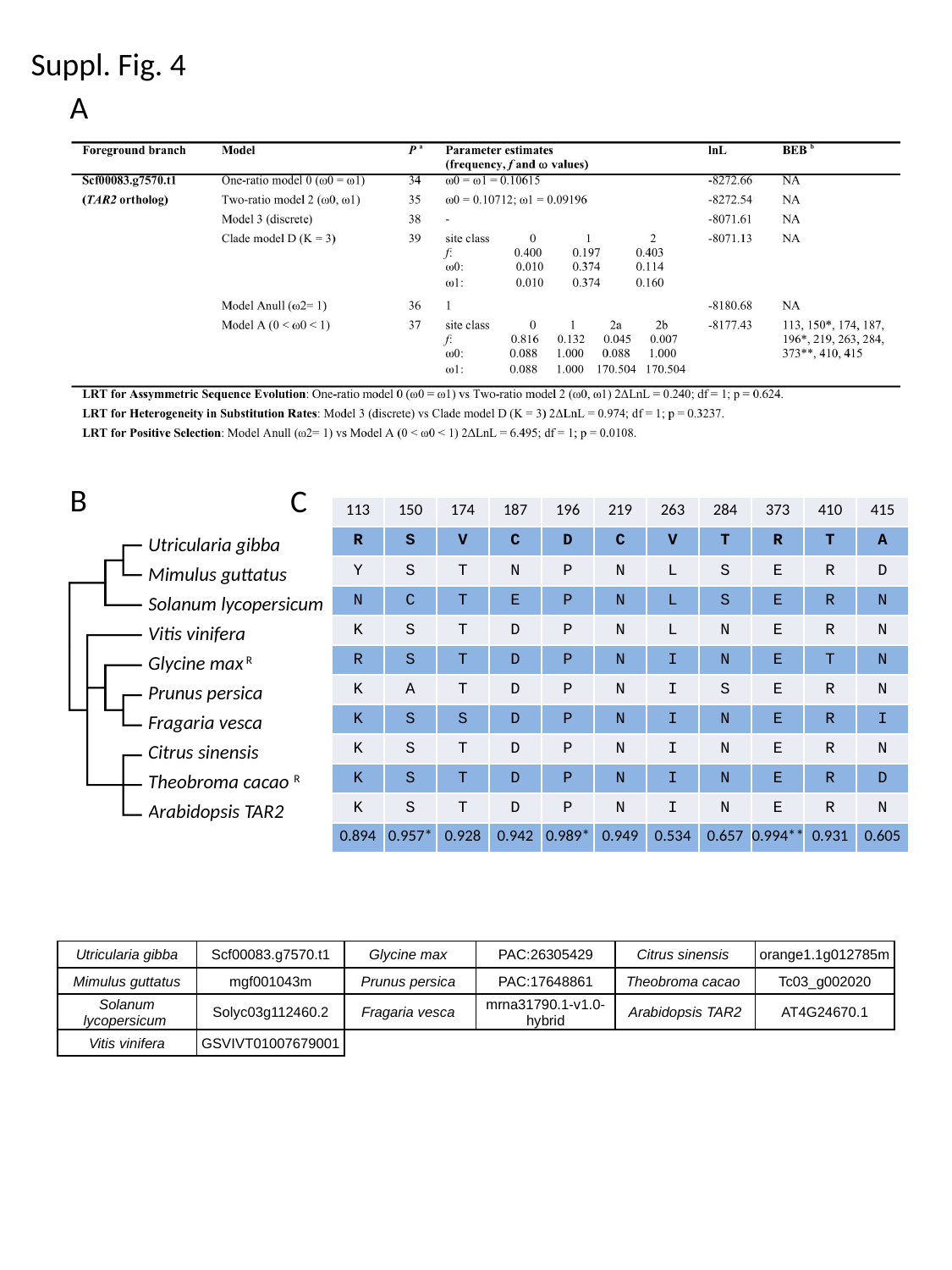

Suppl. Fig. 4
A
B
C
| | 113 | 150 | 174 | 187 | 196 | 219 | 263 | 284 | 373 | 410 | 415 |
| --- | --- | --- | --- | --- | --- | --- | --- | --- | --- | --- | --- |
| Utricularia gibba | R | S | V | C | D | C | V | T | R | T | A |
| Mimulus guttatus | Y | S | T | N | P | N | L | S | E | R | D |
| Solanum lycopersicum | N | C | T | E | P | N | L | S | E | R | N |
| Vitis vinifera | K | S | T | D | P | N | L | N | E | R | N |
| Glycine max R | R | S | T | D | P | N | I | N | E | T | N |
| Prunus persica | K | A | T | D | P | N | I | S | E | R | N |
| Fragaria vesca | K | S | S | D | P | N | I | N | E | R | I |
| Citrus sinensis | K | S | T | D | P | N | I | N | E | R | N |
| Theobroma cacao R | K | S | T | D | P | N | I | N | E | R | D |
| Arabidopsis TAR2 | K | S | T | D | P | N | I | N | E | R | N |
| | 0.894 | 0.957\* | 0.928 | 0.942 | 0.989\* | 0.949 | 0.534 | 0.657 | 0.994\*\* | 0.931 | 0.605 |
| Utricularia gibba | Scf00083.g7570.t1 | Glycine max | PAC:26305429 | Citrus sinensis | orange1.1g012785m |
| --- | --- | --- | --- | --- | --- |
| Mimulus guttatus | mgf001043m | Prunus persica | PAC:17648861 | Theobroma cacao | Tc03\_g002020 |
| Solanum lycopersicum | Solyc03g112460.2 | Fragaria vesca | mrna31790.1-v1.0-hybrid | Arabidopsis TAR2 | AT4G24670.1 |
| Vitis vinifera | GSVIVT01007679001 | | | | |

## Slide 5
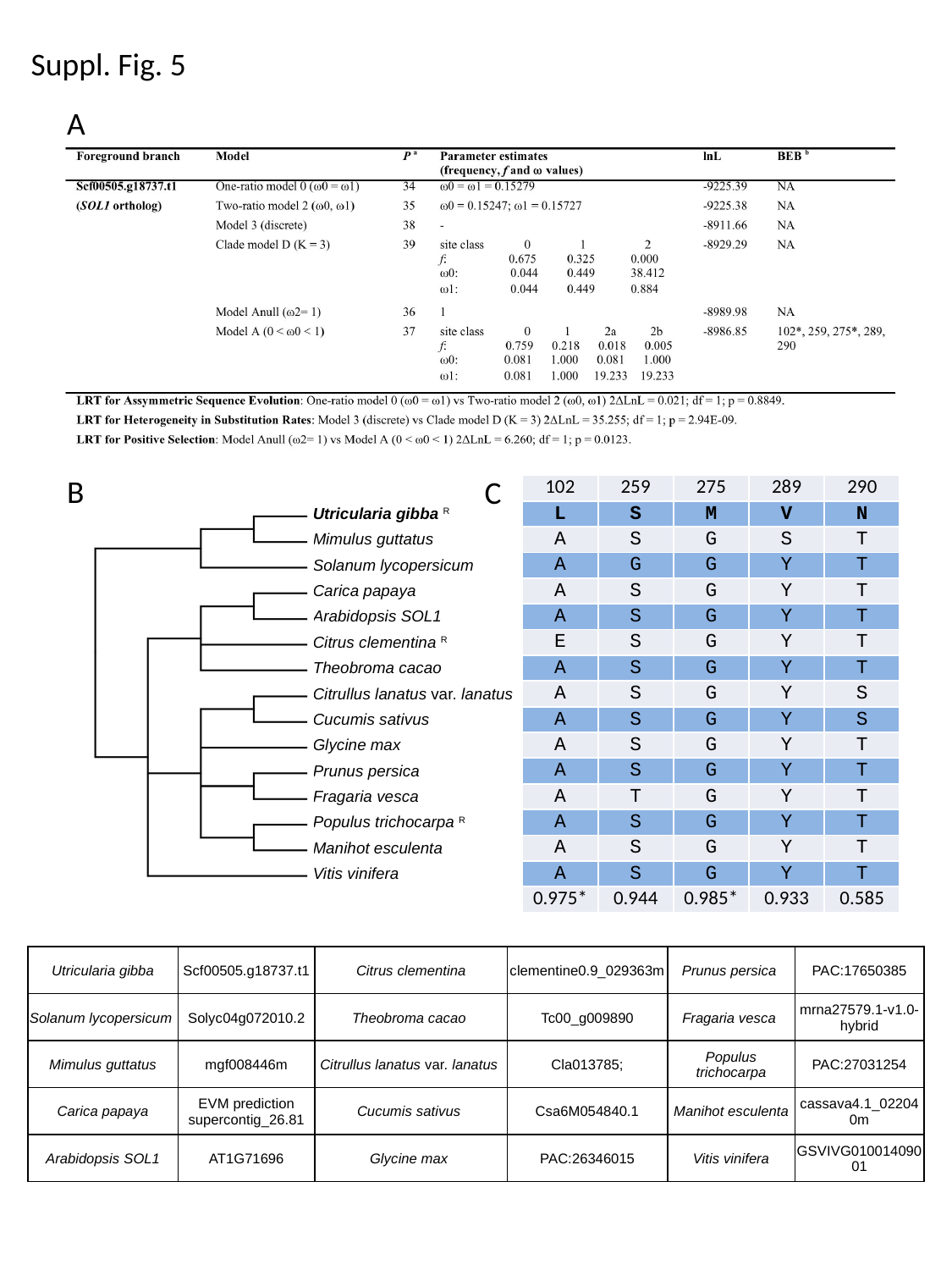

Suppl. Fig. 5
A
B
C
| | 102 | 259 | 275 | 289 | 290 |
| --- | --- | --- | --- | --- | --- |
| Utricularia gibba R | L | S | M | V | N |
| Mimulus guttatus | A | S | G | S | T |
| Solanum lycopersicum | A | G | G | Y | T |
| Carica papaya | A | S | G | Y | T |
| Arabidopsis SOL1 | A | S | G | Y | T |
| Citrus clementina R | E | S | G | Y | T |
| Theobroma cacao | A | S | G | Y | T |
| Citrullus lanatus var. lanatus | A | S | G | Y | S |
| Cucumis sativus | A | S | G | Y | S |
| Glycine max | A | S | G | Y | T |
| Prunus persica | A | S | G | Y | T |
| Fragaria vesca | A | T | G | Y | T |
| Populus trichocarpa R | A | S | G | Y | T |
| Manihot esculenta | A | S | G | Y | T |
| Vitis vinifera | A | S | G | Y | T |
| | 0.975\* | 0.944 | 0.985\* | 0.933 | 0.585 |
| Utricularia gibba | Scf00505.g18737.t1 | Citrus clementina | clementine0.9\_029363m | Prunus persica | PAC:17650385 |
| --- | --- | --- | --- | --- | --- |
| Solanum lycopersicum | Solyc04g072010.2 | Theobroma cacao | Tc00\_g009890 | Fragaria vesca | mrna27579.1-v1.0-hybrid |
| Mimulus guttatus | mgf008446m | Citrullus lanatus var. lanatus | Cla013785; | Populus trichocarpa | PAC:27031254 |
| Carica papaya | EVM prediction supercontig\_26.81 | Cucumis sativus | Csa6M054840.1 | Manihot esculenta | cassava4.1\_022040m |
| Arabidopsis SOL1 | AT1G71696 | Glycine max | PAC:26346015 | Vitis vinifera | GSVIVG01001409001 |

## Slide 6
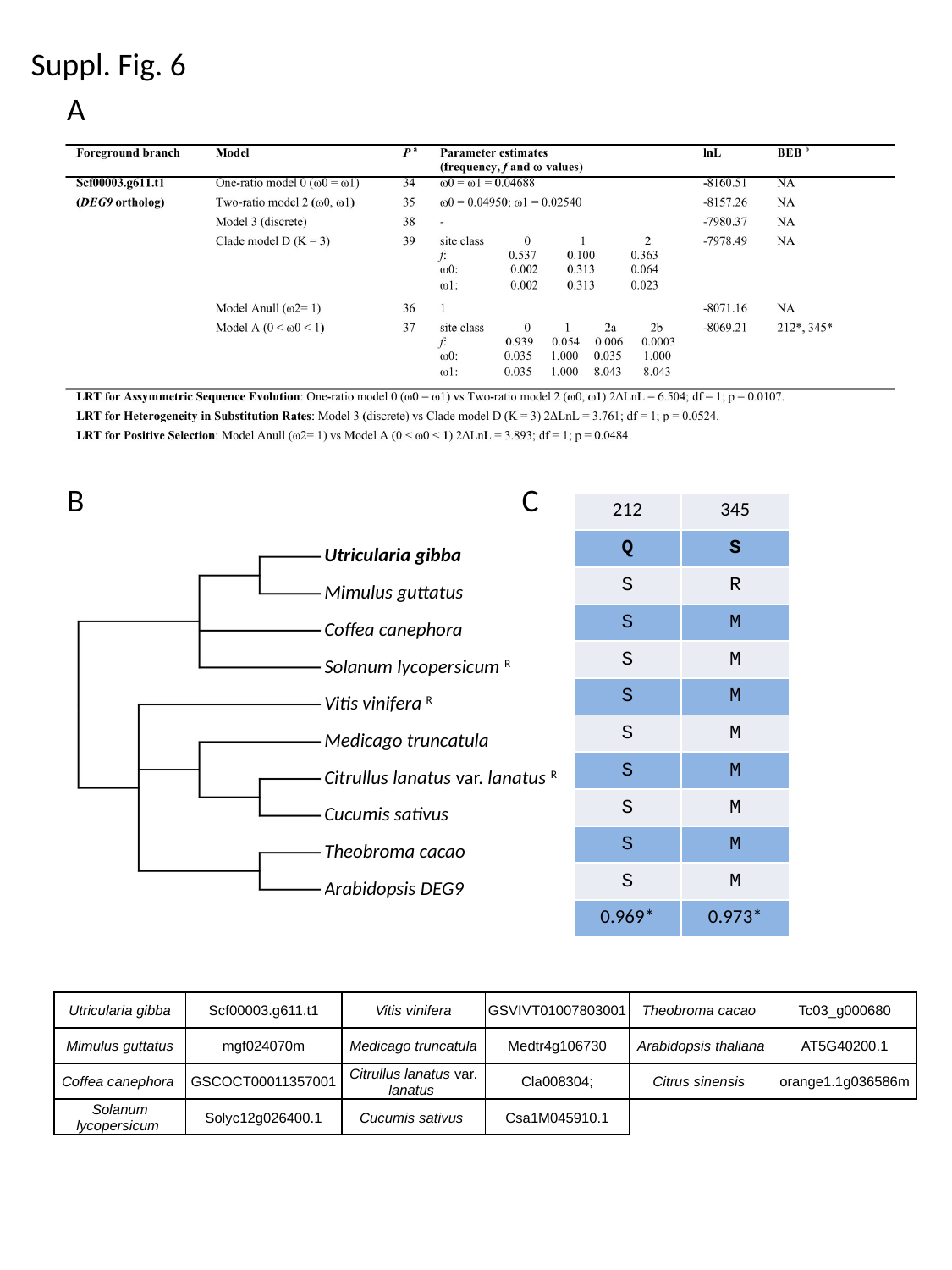

Suppl. Fig. 6
A
B
C
| | 212 | 345 |
| --- | --- | --- |
| Utricularia gibba | Q | S |
| Mimulus guttatus | S | R |
| Coffea canephora | S | M |
| Solanum lycopersicum R | S | M |
| Vitis vinifera R | S | M |
| Medicago truncatula | S | M |
| Citrullus lanatus var. lanatus R | S | M |
| Cucumis sativus | S | M |
| Theobroma cacao | S | M |
| Arabidopsis DEG9 | S | M |
| | 0.969\* | 0.973\* |
| Utricularia gibba | Scf00003.g611.t1 | Vitis vinifera | GSVIVT01007803001 | Theobroma cacao | Tc03\_g000680 |
| --- | --- | --- | --- | --- | --- |
| Mimulus guttatus | mgf024070m | Medicago truncatula | Medtr4g106730 | Arabidopsis thaliana | AT5G40200.1 |
| Coffea canephora | GSCOCT00011357001 | Citrullus lanatus var. lanatus | Cla008304; | Citrus sinensis | orange1.1g036586m |
| Solanum lycopersicum | Solyc12g026400.1 | Cucumis sativus | Csa1M045910.1 | | |

## Slide 7
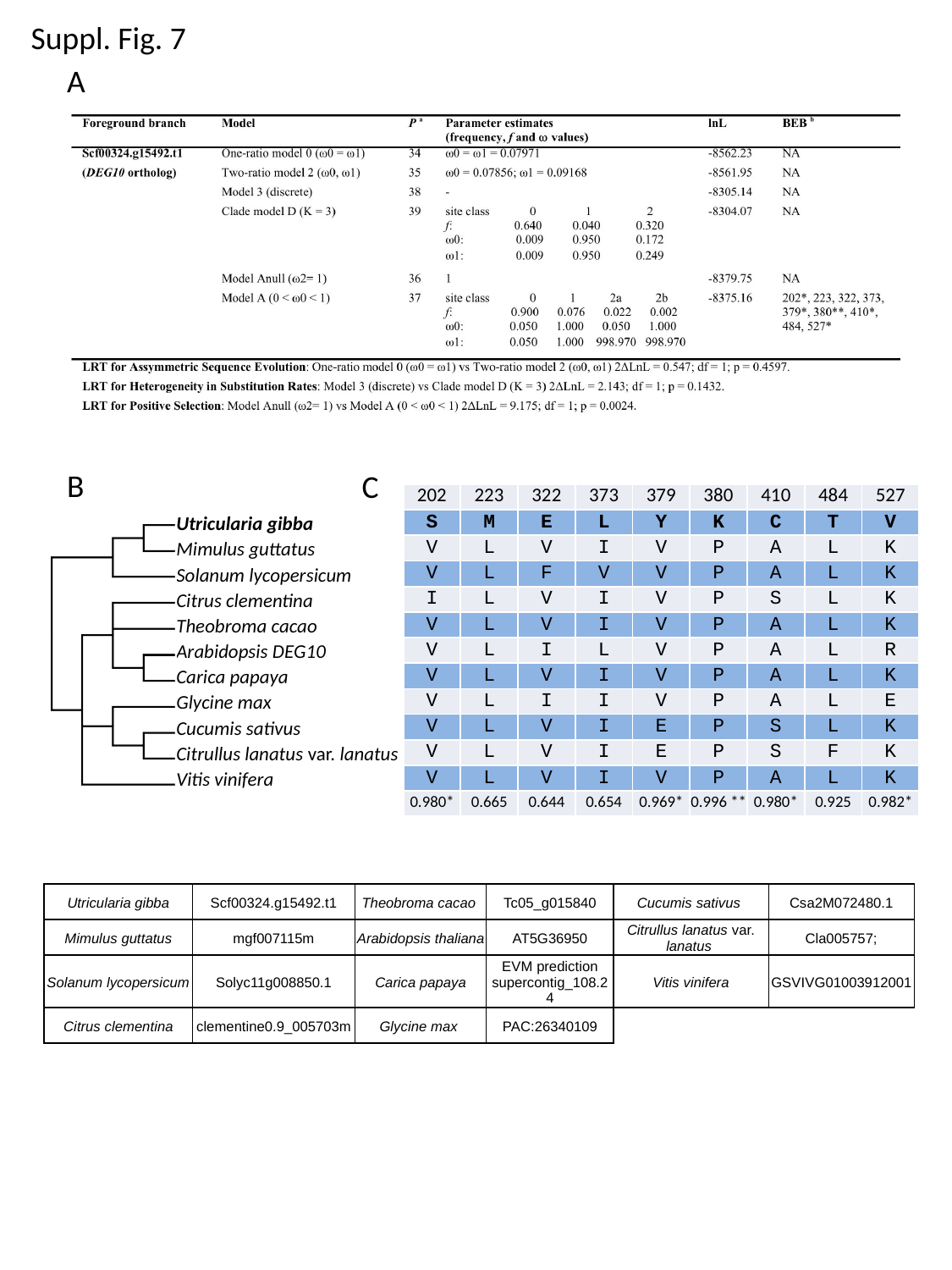

Suppl. Fig. 7
A
B
C
| | 202 | 223 | 322 | 373 | 379 | 380 | 410 | 484 | 527 |
| --- | --- | --- | --- | --- | --- | --- | --- | --- | --- |
| Utricularia gibba | S | M | E | L | Y | K | C | T | V |
| Mimulus guttatus | V | L | V | I | V | P | A | L | K |
| Solanum lycopersicum | V | L | F | V | V | P | A | L | K |
| Citrus clementina | I | L | V | I | V | P | S | L | K |
| Theobroma cacao | V | L | V | I | V | P | A | L | K |
| Arabidopsis DEG10 | V | L | I | L | V | P | A | L | R |
| Carica papaya | V | L | V | I | V | P | A | L | K |
| Glycine max | V | L | I | I | V | P | A | L | E |
| Cucumis sativus | V | L | V | I | E | P | S | L | K |
| Citrullus lanatus var. lanatus | V | L | V | I | E | P | S | F | K |
| Vitis vinifera | V | L | V | I | V | P | A | L | K |
| | 0.980\* | 0.665 | 0.644 | 0.654 | 0.969\* | 0.996 \*\* | 0.980\* | 0.925 | 0.982\* |
| Utricularia gibba | Scf00324.g15492.t1 | Theobroma cacao | Tc05\_g015840 | Cucumis sativus | Csa2M072480.1 |
| --- | --- | --- | --- | --- | --- |
| Mimulus guttatus | mgf007115m | Arabidopsis thaliana | AT5G36950 | Citrullus lanatus var. lanatus | Cla005757; |
| Solanum lycopersicum | Solyc11g008850.1 | Carica papaya | EVM prediction supercontig\_108.24 | Vitis vinifera | GSVIVG01003912001 |
| Citrus clementina | clementine0.9\_005703m | Glycine max | PAC:26340109 | | |
