# Supplementary material for: Genome-Wide Analysis of Adaptive Molecular Evolution in the Carnivorous Plant Utricularia gibba
Source: Genome Biol Evol. 2015 Jan 9;7(2):444–56. doi: 10.1093/gbe/evu288 (PMC4350169; doi:10.1093/gbe/evu288)
Supplement: Supplementary Data [file supp_evu288_suppl_data.zip › New Microsoft Office Word Document.docx]

**Supplementary material**

**Supplementary Table S1: Results of the BadiRate analysis of gene family expansions and contractions:** The fit of the different branch models of gene turnover to the 17234 orthogroups defined by OrthoMCL is shown. For each gene family, the table shows the fit of seven branch models of gene turnover  (Global Rates -GR, ForeGroundUtricularia, ForeGroundTomato, ForeGroundMimulus, ForeGroundGrape, ForeGroundArabidopsis, Free Rates -FR) to the number of genes in the extant species ("Family sizes" column), including their number of parameters, their likelihoods, and their weighted AICs (wAICs). For each family, the table also reports the best-fit branch model ("best-fit model" column), as well as the support for this best-fit model ("support of best fit model"). Best-fit model supports higher than 2.7 (according to the wAIC criterion) were considered significant (shaded cells). In cases where the best-fit model includes lineage-specific GD rates (i.e., the foreground branch models), the table also reports the inferred net family size change in the focal lineage ("Net Gene Number Change in ForeGround Lineage" column).

**Supplementary Table S2. Generic GO functional categorization of genes belonging to the 626 orthogroups identified as significantly expanded in the BadiRate analyses according to Likelihood Ratio Test or wAIC >2.7 in *U. gibba*.** For each GO term, the table shows the number of GO counts among the *U. gibba*-specific expanded genes families (‘sample’ column), the total number of GO counts in the genome (‘background’ column), the Fisher’s exact test of differential GO term distribution (raw and adjusted p-values, by Benjamini-Hochberg), as well as the direction of the differential representation in the sample (over- or under-represented).

**Supplementary Table S3. Plant GO slim functional categorization of genes belonging to the 626 orthogroups identified as significantly expanded in the BadiRate analyses according to Likelihood Ratio Test or wAIC >2.7 in *U. gibba*.** For each Plant GO slim term, the table shows the number of GO counts among the *U. gibba*-specific expanded genes families (‘sample’ column), the total number of GO counts in the genome (‘background’ column), the Fisher’s exact test of differential GO term distribution (raw and adjusted p-values, by Benjamini-Hochberg), as well as the direction of the differential representation in the sample (over- or under-represented).

**Supplementary Table S4. Generic GO functional categorization of genes belonging to the 628 orthogroups identified as significantly contracted in the BadiRate analyses according to Likelihood Ratio Test or wAIC >2.7 in *U. gibba*.** For each GO term, the table shows the number of GO counts among the *U. gibba*-specific expanded genes families (‘sample’ column), the total number of GO counts in the genome (‘background’ column), the Fisher’s exact test of differential GO term distribution (raw and adjusted p-values, by Benjamini-Hochberg), as well as the direction of the differential representation in the sample (over- or under-represented).

**Supplementary Table S5. Plant GO slim functional categorization of genes belonging to the 628 orthogroups identified as significantly contracted in the BadiRate analyses according to Likelihood Ratio Test or wAIC >2.7 in *U. gibba*.** For each Plant GO slim term, the table shows the number of GO counts among the *U. gibba*-specific expanded genes families (‘sample’ column), the total number of GO counts in the genome (‘background’ column), the Fisher’s exact test of differential GO term distribution (raw and adjusted p-values, by Benjamini-Hochberg), as well as the direction of the differential representation in the sample (over- or under-represented).

**Supplementary Table S6. Raw results of the genome wide codon-substitution based molecular evolutionary analysis of the 6848 orthogroups shared by *U. gibba* and four other eudicot species.** Sequence Id names and p-values resulting from the LRT tests are shown from the different branch- and branch-site evolutionary models implemented (i.e., accounting for AE, Assymetric Evolution; HR, Heterogeneous Substitution Rates; PS, Positive Selection). The estimated ω omegas) values resulting from the branch-specific two-ratios and free-ratios models are shown for each branch used as foreground following the same order as the species listed in columns B, C, D and E, i.e., *U. gibba*, Mimulus, tomato, Arabidopsis and grape, the branch leading to rosid species (Arabidopsis and grape) plus the branch leading to asterid species (*U. gibba*, Mimulus and tomato).

**Supplementary Table S7. List of plant genome annotation versions used in this work.**

**Supplementary Figures 1-7:** **Molecular evolutionary analysis of 7 *U. gibba* candidate genes.** Panels 1-7 correspond to orthologs of the Arabidopsis genes *AXR*, *UMAMIT41*, *IGS*, *TAR2*, *SOL1, FYF*, *DEG9* and *DEG10*, respectively. **A.** Parameter estimates, lnL values and LRTs of PAML codon-substitution evolutionary models applied to the evolutionary diversification of X from *U. gibba*. ^a^, number of parameters in the ω distribution; ^b^, amino acids detected by the BEB analysis as fixed by PS (*probability > 0.95, ** probability > 0.99); df = degrees of freedom; p = probability values. **B.** The taxonomy tree used in PAML analysis. Some coding sequences were found to correspond to incorrect gene models, and these were repredicted using Genewise (indicated with R). **C.** The amino acid residues detected as fixed by PS in *U. gibba*, and their homologous positions in the remaining sequences in the alignment. The ids for each sequence examined are indicated in the table below. The ids from the different sequences used are indicated in the table below.

**Supplementary File 1: GO terms calculated for each of the five plant genomes.**

**Supplementary File 2: Plant GO slim terms asigned to each of the five plant genomes.**

**Supplementary File 3: OrthoMCL orthogroups used in BadiRate analysis of gene family turnover.**
